# Optimization of Polygalacturonase Production from a Newly Isolated *Thalassospira frigidphilosprofundus* to Use in Pectin Hydrolysis: Statistical Approach

**DOI:** 10.1155/2013/750187

**Published:** 2013-12-19

**Authors:** V. P. B. Rekha, Mrinmoy Ghosh, Vijayanand Adapa, Sung-Jong Oh, K. K. Pulicherla, K. R. S. Sambasiva Rao

**Affiliations:** ^1^Department of Biotechnology, R. V. R. & J. C. College of Engineering, Chowdavaram, Guntur, India; ^2^Department of Biotechnology, Acharya Nagarjuna University, Nagarjuna Nagar, Guntur, Andhra Pradesh 522510, India; ^3^Department of Animal Biotechnology, School of Applied Life Science, Faculty of Biotechnology, Jeju National University, Jeju City 690-756, Republic of Korea; ^4^Center for Bioseparation Technology, VIT University, Vellore, Tamil Nadu 632014, India

## Abstract

The present study deals with the production of cold active polygalacturonase (PGase) by submerged fermentation using *Thalassospira frigidphilosprofundus*, a novel species isolated from deep waters of Bay of Bengal. Nonlinear models were applied to optimize the medium components for enhanced production of PGase. Taguchi orthogonal array design was adopted to evaluate the factors influencing the yield of PGase, followed by the central composite design (CCD) of response surface methodology (RSM) to identify the optimum concentrations of the key factors responsible for PGase production. Data obtained from the above mentioned statistical experimental design was used for final optimization study by linking the artificial neural network and genetic algorithm (ANN-GA). Using ANN-GA hybrid model, the maximum PGase activity (32.54 U/mL) was achieved at the optimized concentrations of medium components. In a comparison between the optimal output of RSM and ANN-GA hybrid, the latter favored the production of PGase. In addition, the study also focused on the determination of factors responsible for pectin hydrolysis by crude pectinase extracted from *T. frigidphilosprofundus* through the central composite design. Results indicated 80% degradation of pectin in banana fiber at 20°C in 120 min, suggesting the scope of cold active PGase usage in the treatment of raw banana fibers.

## 1. Introduction

Pectic substances are one of the most abundant as well as widely distributed carbohydrates in nature that are classified into protopectin, pectin, and pectinic acid. The primary function of these is gluing together of the individual cells of the plant. Pectic substances in the middle lamella of plant tissue are to help maintain rigidity, firmness, and structure of plant tissues [1].

Pectic substances are naturally degraded by the pectinolytic enzymes and are commonly known as pectinases. The depolymerization process by pectinase enzyme occurs in two ways, first one is hydrolysis, in which pectinolytic enzymes catalyze the hydrolytic cleavage with the introduction of water across the oxygen bridge and the other being transelimination lysis, in which pectinolytic enzymes break the glycosidic bond by a transelimination reaction without the participation of the water molecule. Pectinases act as carbon recycling agents in nature by degrading pectic substances to saturated and unsaturated galacturonans, which are further catabolized to 5-keto-4-deoxy-uronate and finally to pyruvate and 3-phosphoglyceraldehyde [2].

Pectolysis is one of the most important processes in plant cell elongation, growth, and fruit ripening. They act on the galacturonan part of the pectin molecule to produce smaller fragments, disrupting the rigidity of the tissue or the consistency of the juice or puree. They also help in the cell wall extension and softening of some plant tissues during maturation and storage.

Paper and food industries rely on the usage of pectinase isolated from the microorganisms. Fruit juices and wine industries use the enzyme to facilitate the expression for higher yield of juice and clarify wines, while in paper industries the enzyme is utilized for degumming [3, 4]. Another commercial application includes tea and coffee fermentation, olive oil recovery, vegetable oil extraction, textile industry, and animal feeds [5].

Microorganisms of extreme temperature have gained more scientific attention because of their importance in biotechnological processes. The application of the mesophilic enzymes is restricted due to their limited stability at the extremes of temperature, pH, and ionic strength. Due to this limitation, the cold active enzymes can play a significant role over mesophilic and thermophilic enzymes. High specific activity at low temperature, saving of labile or volatile compounds, reducing the risk of mesophilic and thermophilic contamination, energy saving, and easy inactivation of enzymes during postprocessing are steps to attract the attention of industries to treat their products with these cold active enzymes [6].

The present work was conducted to screen the production of pectinolytic enzymes from the psychrophilic bacterium. The activity of pectinolytic enzymes, namely, pectatelyase (PGLase), pectin lyase (PLase), polygalacturonase (PGase), and polymethylgalacturonase (PMGase) was quantified. The study was also conducted to optimize all the parameters influencing PGase production.

In general, the optima is carried out by one factor at a time, but in case of a large number of variables, the one-factor-at-a-time method becomes tedious and time consuming and usually does not lead to the exact determination of optimal conditions. Optimization through Taguchi design and response surface analysis is a common practice for optimization process [7]. The present study was conducted to optimize the parameters influencing the PGase production by the sequential statistical approach using Taguchi and CCD of RSM.

Furthermore, the artificial neural networks (ANN) can be useful to improve the accuracy level by training it on experimental data [8]. ANN is organized in three groups, or layers: input, hidden, and output. The sum of input at each neuron in the hidden layers and the sum of the output from the hidden layers process the result of neuron's network function. Many of the most recent techniques use genetic programming to develop ANNs. Genetic algorithm (GA) based on the evolutionary method of natural selection of the best individuals in a population is commonly used to optimize a given function over a certain range. ANN is used as fitness function of the genetic algorithm (GA) in order to determine the optimum parameters of medium components for maximum PGase production. Media constituents thus optimized by statistical experimental design were analyzed by ANNs and comparative analysis between ANN and RSM was conducted.

The crude pectinase extracted from *T. frigidphilosprofundus* was investigated for its ability of banana fiber hydrolysis. The pseudostem of the banana plant is usually considered as a waste in the banana industry which makes sense to turn this waste into a useful product like paper. The pseudostem has high content of holocellulose and low percentage of ash and lignin, which is suitable for pulping in paper industry [9]. But, the presence of pectin and lignin leads to crumbling and dullness of paper. Thus, it is important to remove lignin and pectin before processing the paper to achieve better quality [10]. The effect of temperature and incubation time as well as the effect of crude enzyme on pectin degradation from banana fibers was investigated and optimum level of influencing factors was standardized by RSM. To the best knowledge of the author, this is the first report on the optimization of cold active PGase production from the bacterium *Thalassospira* sp. and the enzyme being further used for pectin hydrolysis in banana fiber.

## 2. Material and Methods

### 2.1. Chemicals

Polygalacturonic acid, the substrate for PGase and PGLase and pectin as the substrates used for PLase and PMGase were procured from Sigma Aldrich, India. Marine Agar 2216 was purchased from Difco. All other chemicals used in this study were of analytical grade procured from Hi media, India.

### 2.2. Microorganism and Maintenance

Marine cold adaptive bacterium *T. frigidphilosprofundus* was used for all experiments. The cell morphology was studied using scanning electron microscope. The isolate was identified as *T. frigidphilosprofundus* (98% sequence identity) based on 16S rRNA gene [11] and the strain is deposited in the National Center for Industrial Microorganisms, Pune, India, with accession number 5438. The culture was maintained on Marine agar at 20°C and subcultured for every 14 days interval.

### 2.3. Plate Assay to Determine the Depolymerization of Pectin

The pectinolytic activity of novel cold adaptive strain was detected by plate method. The bacterial sample was loaded onto cork bored well of pectin marine agar media plates containing (per liter) 2.5 g of citrus pectin, 5 g of bactopeptone, 12 g of sea salt, 0.1 g ferric citrate, 1.8 g calcium chloride, 5.9 g magnesium chloride, 0.55 g potassium chloride, 0.034 g strontium chloride, 0.022 g boric acid, 0.004 g sodium silicate, 0.0024 g sodium fluoride, 0.0016 g ammonium nitrate, 0.008 g di-sodium phosphate, 0.08 g potassium bromide, and 15 g of agar (pH 6.5) incubated at 20°C for 4 days. When the colonies reach around 5 mm diameter, the culture plates were flooded with freshly prepared 0.005% of ruthenium red solution and allowed to stand for 30 minutes.

### 2.4. Production Medium and Culture Condition

A loopful of the strain from pectin marine agar plates was transferred to 100 mL sterile pectin marine broth. The pH of the medium was adjusted to 7.0 and the culture was incubated on a rotary shaker at 150 rpm and 20°C. The seed culture was allowed to grow for 30 h and then 5% of the seed culture was transferred to production medium containing (per liter) 5 g peptone, 0.1 g ferric citrate, and 5.9 g magnesium chloride, 14 g sodium chloride, 1.8 g calcium chloride, 5 g pectin and pH was adjusted to 7.6 ± 0.2 and incubated at 20 ± 2°C for 48 h.

### 2.5. Enzyme Assays

The enzyme assays were conducted based on protocol adapted from the literature, considering several variables [12–14]. For enzyme assay, two mL of freshly grown culture was taken and centrifuged at 10,000 rpm for 10 min. The supernatant (100 *μ*L) from the culture broth was served as the source of the enzyme. PLase and PGLase activities were assayed at 235 nm by measuring the formation of unsaturated oligogalacturonates. PLase assay mixture consisted of 0.25% (w/v) pectin in 100 mM citrate phosphate buffer (pH 6.4). PGLase activity was tested by replacing pectin with 0.15% (w/v) of polygalacturonic acid (pH 7.5). The reaction was stopped by adding 3 mL of 0.5 M HCl. The assay mixture was incubated for 20 min at 20°C and the absorbance was measured at 235 nm against the suitable blank. The molar extinction coefficient of 5550 M^−1^ mL^−1^ was applied to calculate the enzyme activity. One unit was defined as the amount of enzyme that released one *μ*mol mL^−1^ min^−1^ of unsaturated oligogalacturonates at 235 nm per minute under the assay conditions.

PGase was assayed with PGA (polygalacturonic acid) which acted as a substrate. The assay mixture was prepared with the following components: 0.2 mL enzyme, 0.1 mL of 0.1 M CaCl_2_, and 0.5 mL of 1% solution of PGA (1.0 g of PGA in 100 mL of 0.1 M citrate buffer, pH 6.6). The reaction mixture was incubated at 20°C for 30 min. The reaction was terminated by adding 0.5 mL of copper reagent and boiled for 10 min. After cooling to room temperature, 0.5 mL of arsenomolybdate reagent was added to the assay solution. The absorbance was read on a spectrophotometer at 500 nm against suitable blank. For PMGase assay, 86 *μ*L of culture filtrate was used. The assay was conducted by same procedure described for PG but pectin (0.25%, pH 5.3) is used as a substrate instead of polygalacturonic acid (pH 6.6, 20°C). A molar extinction coefficient of 212.12 M^−1^ mL^−1^ was used to calculate the enzyme activity. One unit of enzyme activity is defined as the enzyme that releases 1 *μ*mol mL^−1^ min^−1^ of galacturonic acid under standard assay conditions.

### 2.6. Effect of NaCl and Natural Pectic Substrate on PGase Activity and Biomass

The effect of NaCl concentration on biomass and PGase production was studied by inoculating the strain in production medium that contained 5.00 gm/lt to 20 gm/lt of NaCl. The optical density of the culture was measured at 600 nm. To study the effect of natural pectic substrate on PGase activity and biomass production, citrus pectin was substituted with orange and apple peel (0.25%) in the production medium.

### 2.7. Influence of Temperature, pH, and Incubation Time on PGase Production

The influence of initial pH and temperature on PGase production by *T. frigidphilosprofundus* was studied. The pH of the media was adjusted with 0.5 M NaOH. The effect of pH on the activity and stability of PGase was investigated at different pH values ranging from 3 to 9. Enzyme solutions were mixed with the buffer solutions and incubated at 20°C. Under standard assay conditions the aliquots of the mixtures were taken to measure PGase activity. The effect of incubation time on PGase was determined under standard conditions with different time intervals ranging from 0 to 60 min. The effect of temperature on the activity of PGase was determined using a wide range of temperatures from 4 to 50°C and the thermal stability was investigated by measuring the residual activity after incubating the enzyme with the previously mentioned temperature ranges.

### 2.8. Experimental Design

Statistical methods Taguchi L_27_ orthogonalarray (L_27_OA) and RSM were used for optimization of media components. The media optimized by L_27_OA method was used as an input for CCD for final optimization. All the experiments were performed in triplicates.

### 2.9. Optimization of PGase Production by Taguchi L_**27**_ Orthogonal Array Method

L_27_OA in Taguchi methodology was employed for screening the most influential fermentation parameters effecting PGase production by *T. frigidphilosprofundus*. Minitab 13.0 (Minitab Inc., PA, USA) was used for the experimental designs and subsequent regression analysis of the experimental data. Each independent variable was tested at three levels such as high, middle, and low level. The symbol code and actual level of the variables and the experimental design are shown in Tables 1 and 2. Six factors such as orange peel, bactopeptone, MgCl_2_, NaCl, CaCl_2_, and ferric citrate variables were studied in 27 experiments to calculate the standard error. The triplicate verification test was performed to check the optimum condition and the average value was taken as the response. The variables in confidence levels above 90% are considered to have a significant effect on PGase production and thus the selected crucial factors have been used for further optimization by RSM.

### 2.10. Optimization of the Selected Medium Components by RSM

RSM is one of the widely used statistical approaches for final optimization of process parameters which plays an important role in designing experiments (DOE). The level of independent factors in RSM was increased or decreased according to the results of L_27_OA. In general, for RSM design, each factor is to be tested at three levels. The lower, middle, and high levels of each variable were designated as −2, −1, 0, 1, and 2, respectively.

To examine the combined effect of the independent variables on PGase production, CCD was employed. According to this design, the total number of treatment combinations is 2*k* + 2*k* + *n*_*o*_, where “*k*” is the number of independent variables and *n*_*o*_ is the number of repetitions of the experiments at the center point. A total of 30 experiments, including six center points, were carried out. The replicates of center point were used to evaluate the pure error and also to verify any changes in the estimated model.

A quadratic polynomial regression model was assumed to predict response. The relationship of the independent variables and the response was calculated by the second-order polynomial and expressed in the form of a quadratic model equation

(1) $$\begin{array}{c} \text{SE}=\pm \sqrt{v\left(b\right)ii}, \end{array}$$

where *v*(*b*) is variance-covariance matrix and *i* = 1,2,…, *n*, are the variances of the regression parameters given in the same order as mentioned in the regression equation.

### 2.11. Data Analysis by ANOVA

Experimental designs and the polynomial coefficients were calculated and analyzed using Design-Expert software (version 6.0.10, Stat-Ease Inc., Minneapolis, USA).

The analysis of the variance (ANOVO) was used to determine significance of variables. The model fitness was analysed base on the Regression Coefficients (*R*²), Adj *R*-Squared (Adj *R*²), Pred *R*-Squared (Pred *R*²), lack of fit. Regression value compares the accuracy of the model. The  *R* ^2^ is mathematically described as

(2) $$\begin{array}{c} R²=1-\frac{\sum_{i=1}^{1-n}{\left(y_{i}-y_{i}^{\prime }\right)}^{2}}{{\sum_{i=1}^{1-n}\left(y_{i}-y\right)}^{2}}, \end{array}$$

where *n* is the total number of variables, *y*_*i*_ is the actual value, *y*_*i*_′ is the value predicted by the network, and *y* is the mean of *y* values.

In order to validate the model, all the experiments were conducted at triplicates. Experiments were carried with optimal levels of all parameters and averages of the results were taken as a response.

### 2.12. Optimization of Medium Components Using ANN-GA

In ANN model, the output of the neuron largely depends on the transfer functions. It is also called as activation function for the ANN model which is three types in feed forward backpropagation neural network. The feed forward backpropagation neural network of ANN was used to build predictive model with concentrations of four media components as an input and yield of PGase production as an output of the model. The present study consists of ten artificial neurons at three hidden levels. In network, only the number of nodes in hidden layer can be specified (layer 1) while the number of node of the output layer (layers 2 and 3) depends on the number of target output data. The weights assigned are indicated with each arrow, which also represent the flow of information. These weights are multiplied by the values which go through each arrow, to give more or less strength to the signal which they transmit. In neurons, the output layers receive the data from respective weights of input neurons. Once the neural network was created, it was trained to accurately model the given phenomenon using the experimental data.

An artificial neuron's network consists of an interconnected group of artificial neurons, and it processes information using a connectionist approach to computation. The model in ANN is referred to as a threshold unit which receives input from other units denoted as *N*.

Now, if the previous output of the neuron (weighted sum of the inputs) is greater than the threshold *t* of the neuron, then the output of the unit would be 1 and 0 otherwise. Thus, the output can be expressed as

(3) $$\begin{array}{c} O=g\left(\overset{N}{\underset{i=1}{\sum }}w_{1}x_{1}-t\right), \end{array}$$

where “*g*” is the step function, which is 0 when the argument is negative and 1 when the argument is not negative.

The training function of feed forward backpropagation neural network was applied to minimize the prespecified error function by adjusting the weights appropriately. The mean standard error (MSE) and the mean absolute error (MAE) were used as measures of closeness of the model to the actual system.

Once ANN model has developed, the GA was used to optimize the maximum possible PGase production. The GA parameters chosen for this study were population size = 50, the maximum number of generations = 1000, crossover probability = 0.95, mutation probability = 0.04, total string length = 20, and the number of binary coded variables = 4. The maximum and minimum limit of the factors for GA was between the levels specified at RSM design (Table 6). The GA for the maximum production of PGase can be expressed as

(4) y=f(X,W),  xiL≤xi≤xiU, i=1,2,…,P,

where *X* denotes the input vectors, *W* is expressed as corresponding weight of vectors, *f* represents the objective function of ANN model and *y* refers to the production of PGase, *x* denoted the factors, and *x*_*i*_^*L*^ and *x*_*i*_^*U*^ are the lower and upper limit of the *x*_*i*_.

### 2.13. Estimation of Pectin Hydrolysis in Treated Banana Pseudostem

In the present study, the effect of pectinase enzyme on pectin hydrolysis from banana fiber was determined by estimating the reducing sugars and the total pectin concentration in the test sample.

The shredded and dried fiber from banana pseudostem was used for the experiment. The reducing sugar level was checked in the treated banana fiber by DNS method [15, 16]. The absorbance at 540 nm was measured using ELICO SL-159 UV-Vis spectrophotometer. The effect of enzyme treatment on banana fiber was visualized by scanning electron micrographs.

Simultaneously, the pectin concentration is also measured in the test sample to determine the enzyme activity. The pectin extraction procedure was based on the acid hydrolysis of protopectin. The crude enzyme treated banana fiber was collected from each trial and washed with the distillated water three times. The macerated sample has been taken with 250 mL of distilled water with a pH between 1.2 and 2.6 (adjusted with 0.5 M HCl), followed by continuous stirring for 20 min at 90°C. The residue was collected in the first step and again transferred into 250 mL of acid water (pH 2.6, adjusted with 0.3 M HCl) for 10 min at 90°C. The filtrates were pooled and plunged in ice to stop the hydrolysis process. The supernatants were recovered after being filtered on a grid and then frozen. To avoid the pectin hydrolysis the pH of HCl extract was neutralized by 0.5 mL of 1 N NaOH. The pectin was precipitated with two volumes of ethanol for one volume of supernatant. The obtained precipitate was washed with 6.6% ethanol and centrifuged (10,000 rpm during 20 min). The solution was then filtered using fine filter cloth to separate jelly pectin and later dried under vacuum at 50°C.

The pectin yield (%) is calculated using the following equation:

(5) $$\begin{array}{c} \%\;\text{of}\;\text{pectin}\;\text{yield}=\left(\frac{\text{amount}\;\text{of}\;\text{pectin}\;\text{extracted}}{\text{wt}.\;\text{of}\;\text{sample}\;\text{for}\;\text{estimation}}\right)100. \end{array}$$

### 2.14. Optimization of the Parameters for Pectin Hydrolysis in Banana Fiber

Optimization of the physical and chemical factors for pectin hydrolysis in banana fiber was carried out by RSM. It provides statistically validated predictive models that can be applied for finding optimal process configurations. In this study, the effects of four components, namely, volume of crude enzyme used, temperature, amount of banana fiber, and incubation time, have been taken as independent variables on pectin hydrolysis. In CCD, total of 30 experiments were performed including 6 central points. All the mentioned independent variables were designed at five different levels; zero was considered as the center coded value. The relation among the variables, that is, crude enzyme extract used in the experiment, temperature, incubation time, and amount of the banana fiber used in the experiment, was explained in terms of a quadratic model using (4).

### 2.15. Data Analysis and Validation of the Model

Experimental designs and the polynomial coefficients were calculated and analyzed using Design-Expert (version 6.0.10, Stat-Ease Inc., Minneapolis, USA) and the combined effect of the factors on pectin hydrolysis were visualized by three dimensional graphs constructed with the aid of Sigma Plot. The validation of the model was carried out by conducting experiments in triplicates with the optimum level of the variables and the mean values were tabulated.

## 3. Results

**Figure 1:**
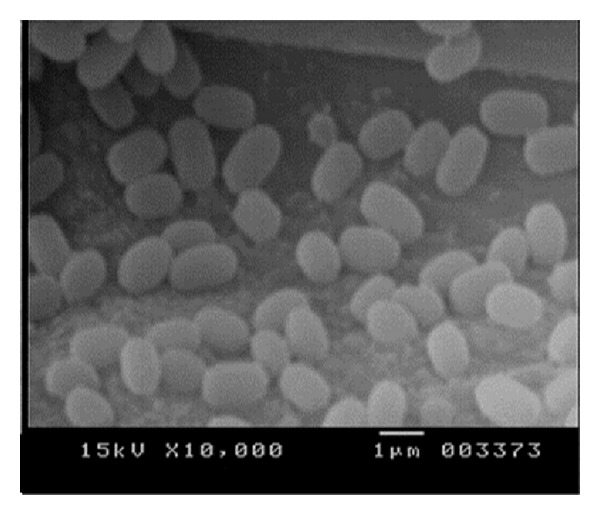
The figure illustrated the scanning electron micrographs of banana fiber. The pictures were taken before (a) and after (b) treatment with crude pectinase.

The deep marine cold adaptive strain used in the present study was isolated from Bay of Bengal and it is the first reported *Thalassospira* strain from that region (Figure 1). Primary screening of the isolated strain was performed on pectin-agar plates, where pectin was the sole carbon source for the growth of the organisms. It was observed that *T. frigidphilosprofundus* was capable of utilizing pectin. The clear halo zone around the colonies was visualized, indicating the hydrolysis of pectin. Initially the activities of PLase and PGLase were determined as 0.8 and 0.5 U/mL, whereas the activity of PGase and PMGase was found at 3.00 U/mL and 2.06 U/mL, respectively. The result clearly showed that maximum activity was found in PGase; therefore as per the objective, the study has been carried out to optimize the production of PGase from bacterium *T. frigidphilosprofundus*.

**Figure 2:**
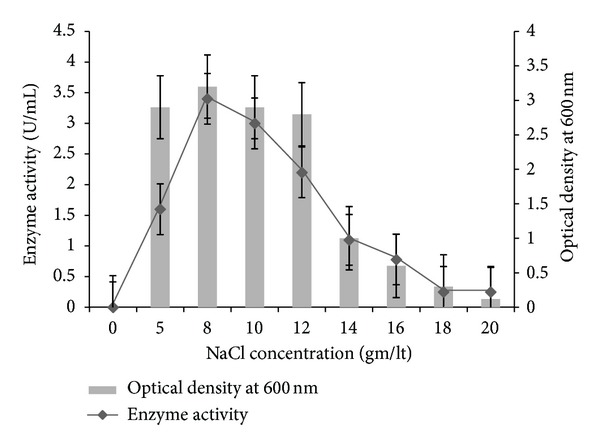
The figure showed the effect of NaCl concentration on biomass and PGase activity.

To study the effect of NaCl concentration on biomass and PGase production, the isolate *T. frigidphilosprofundus* was grown at different concentrations of NaCl in the media.  Figure 2 shows the influence of NaCl on biomass and PGase activity. It has been observed that the growth rate of *T. frigidphilosprofundus* was not affected up to 12 gm/lt NaCl concentration and thereafter the growth decreased. The highest PGase activity (3.62 U/mL) was found at 8 gm/lt NaCl concentration.

**Figure 3:**
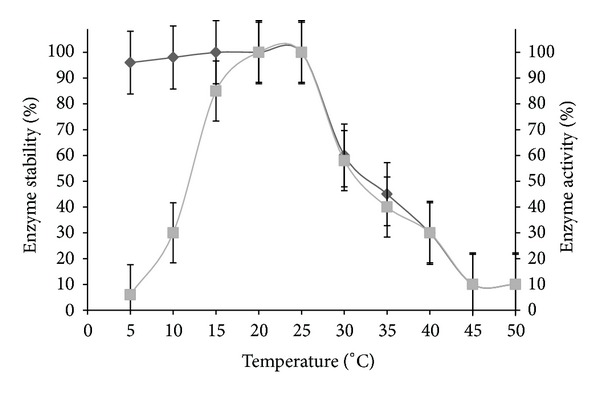
The figure has shown the effect of temperature on PGase activity and its stability.

**Figure 4:**
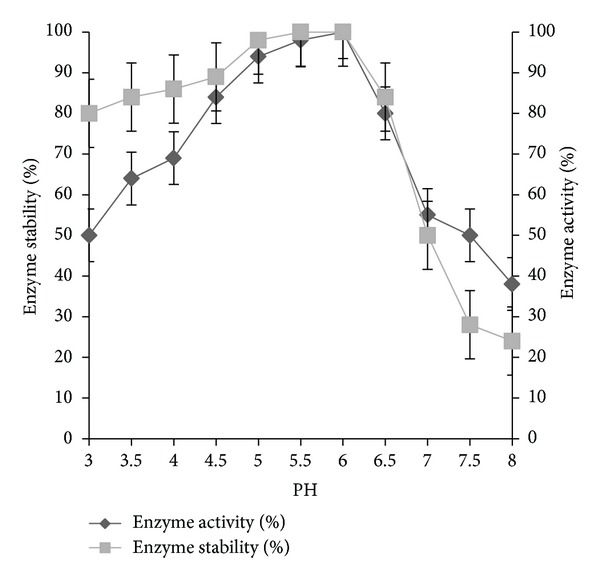
The figure illustrated the effect of pH on PGase activity and its stability.

The highest biomass and PGase activity was found at 20°C, pH 6. The strain had shown 40% of PGase activity at 5 to 15°C, and the thermal stability of the enzyme was found between 0 and 35°C (Figure 3). The pH of the enzyme activity is relatively wide. The optimum pH of PGase was determined as 6. The activity was stable over the pH range of 4.5 to 7.5 at 20°C, and 80% activity was found between the pH ranges of 3.0 and 5.5 (Figure 4). The influence of incubation time on PGase revealed that the enzyme activity was gradually decreased with extending the incubation period. The highest PGase activity was found between 20°C to 25°C after 20 min of incubation. The effect of natural pectic substrates on growth and PGase production was demonstrated. Apple peel did not show much influence on growth and PGase production, whereas feeding of orange peel to the production medium increased the activities from 3.00 U/mL to 5.84 U/mL in 30 h of incubation at 20°C.

### 3.1. Optimization of the Factors for PGase Production

The relationship between medium components and their concentrations could be calculated using L_27_ orthogonal array design. The optimal combinations and the concentration of the factors required to achieve the maximum PGase activity were represented in Table 2. The PGase activity was estimated as 16.48 U/mL, which is closely associated with the prediction value.  Table 3 represents the response table for mean and *S/N* ratio to understand the delta and rank value of the system. In the present study, it can be seen that for each of the six variables at three levels, one level increases the mean compared to the other level. Thus the factors, that is, orange peel, bactopeptone, MgCl_2_, and CaCl_2_, have shown a maximum effect at level 3, whereas the highest effect of ferric citrate and NaCl was found at level 1 (Table 3). It represents the optimal concentrations of the individual components in the medium. The interaction plot between the factors was illustrated in the supplementary document (supplementary Figure 1, available online at http://dx.doi.org/10.1155/2013/750187).

The results of mean and *S/N* ratio were tabulated in Table 3. Based on the delta values of *S/N* ratio, the most influencing factors are categorized as orange peel > bactopeptone > NaCl > MgCl_2_ > CaCl_2_ > ferric citrate. The statistical significance was checked by ANOVA (Table 4). According to linear regression model, the response model was established.

The calculated *F* value should be several times greater than tabulated value, thereby the null hypothesis is rejected at *α* level of significance. In this study, the *F* value corresponding to PGase was 155.25, which is greater than the tabulated *P* value at 0.005 level of significance. The coefficient of regression (*R* ^2^) is a measure of total variation of response about the mean found in fitted model. In the PGase production the value of *R* ^2^ indicates that 98.4% sample variation is attributed to the chemical parameters and only 1.6% is not explained by the fitted model. According to the ANOVA analysis *χ*_1_, *χ*_2_, *χ*_3_, and *χ*_6_ were identified as the major influencing factors in the enzyme production and these four factors were considered for further optimization by RSM.

### 3.2. Optimization of the Selected Factors Using CCD

The objective functions in L_27_OA design illustrated the influence of six selected independent factors on production. It was observed that orange peel, bactopeptone, MgCl_2_, and NaCl in PGase has shown the maximum influence, whereas ferric citrate and CaCl_2_ showed less effect on PGase production. Thus, an adjustment in the final concentrations of those four factors should result in a higher production of PGase. The center point of the L_27_OA has been considered as the origin of the path. The levels of each factor and the design matrix are given in Table 5. The predicted and the response values of the model had been given in Table 6. The maximum production of PGase (28 U/mL) was obtained at the trial number 14. This experimental value was found to be nearer to the predicted value of PGase production.

The second-order model equation was quantified by using analysis of variance (ANOVA). In this study it was found that *χ*_2_, *χ*_3_, *χ*_6_, *χ*_1_^2^, *χ*_2_^2^, *χ*_3_^2^, *χ*_1_ *χ*_3_, and *χ*_2_ *χ*_3_ are the model terms. The second-order response model was established after analysis of regression. The model can be shown as follows as final equation in terms of actual factors:

(6) PGase (U/mL) =+24.75−0.19χ1+0.95χ2−0.66χ3  −0.91χ6−1.63χ21∓0.41χ22−0.39χ32  −0.22χ62+0.1χ1χ2+0.97χ1χ3  −0.11χ1χ6+0.52χ2χ3−0.023χ2χ6  −0.044χ3χ6.

The significance of the quadratic regression model was checked by Fisher's test (*F*-test) and ANOVA (Table 7). The *F* value corresponding to PGase was 16.45 with a very low probability value (*P*  model > *F* is 0.0001) which justified the significance of the model. In general, the *F* value should be several times greater than the tabulated *F* value that implies the rejection of the null hypothesis at significant level of *α*. In this study the null hypothesis is rejected at 0.0001 significant level.

The *R* ^2^ was significant at the level of 93.90% in PGase production which infers that only 6.1% of total independent variable was not explained by the model. It indicates that all the independent factors contribute to a combined effect to maximize the production of PGase. The adequate precision measures the signal to noise ratio in the model. This ratio greater than 4 is desirable. In this study, the ratio of 17.759 indicates an adequate signal for the model. Therefore, this model can be used to navigate the design space. The values of “Prob > *F*” less than 0.0500 on the model are considered as significant.  Table 8 represented Student's *t* distribution and the corresponding *P* value along with the coefficient estimate.

The orange peel, bactopeptone, and MgCl_2_ exhibited significant effects on PGase production. The maximum unit of PGase production was found to be 29.39 U/mL. The level of migration and relations between the predicted and experimental values in the PGase production was determined by parity plot. The points around the diagonal line indicated the minimum diversion between the experimental values and predicted data (supplementary Figure 2).

### 3.3. Final Optimization of Medium Components Using ANN-GA

The correlation between the concentrations of medium components and the production of PGase has been resulted in 93% of square regression. In RSM model, the interaction effects of orange peel with MgCl_2_ and bactopeptone with NaCl were found to be significant.

Once the generation capable ANN-based process model with good prediction accuracy is created, the input spaces of the ANN model could be optimized with the objective of maximum PGase production [17]. The PGase activity of ANN was predicted and the MSE and the MAE were found to be 0.0096 and 0.0064, respectively. The ANN-GA result showed the increment of PGase production (31.47 U/mL) (Table 9), which is close to the value predicted by the model. Although both RSM regression model and ANN model provided overall accurate predictions, regression equation in ANN model (*R* ^2^ = 0.9836) showed better correlation with the experimental PGase production than in RSM regression model.

### 3.4. Optimization of Categorical and Numerical Process Parameters for Maximum Pectin Hydrolysis

The reducing sugar level in treated sample was checked at regular intervals to study the effect of enzyme on the pectin degradation from banana fibers. It was interesting to notice that the reducing sugar level was increasing gradually indicating the effectiveness of the treatment. The coded values of independent variables are displayed in Table 10(a).

Experiments were carried out according to the design matrix to optimize the volume of enzyme used, temperature, amount of banana fiber, and incubation time. The experimental results and predicted response are given in Table 10(b). It was found that 15 mL of crude enzyme hydrolyzed 40 mg of banana fiber at 15°C after 120 min incubation. According to the ANOVA of the quadratic model, the model is significant as is evident from Fisher *F* test. In this study *A*, *B*, *D*, *A* ^2^, *B* ^2^, *AB*, *BC*, and *CD* factors for percentage of pectin yield and *A*, *D*, *C* ^2^, *D* ^2^, and *CD* factors for level of reduction sugar were denoted as significant model terms. The square of regression was found to be 0.9840 and 0.8671 for pectin yielded (%) and reducing sugar, respectively. The final second-order coefficient equation for percentage of pectin yielded and reduction sugar level was represented in the term of coded factors as follows:

(7) %  of  Pectin  yeild   =+1.49−0.64A+0.33B+0.060C−0.15D  +0.40A2+0.50B2+0.066C2+0.052D2  −0.041AB+0.017AC−0.071AD  +0.15BC+0.076BD−0.35CD,Reducing  Sugar =+0.12+0.058A−0.015B+1.958C  +0.021D+1.88A2−0.010B2+0.019C2  +0.022D2−0.016AB−1.844AC+2.531AD  −1.844BC−2.46BD+0.025CD.

Taken all together in order to achieve the maximum pectin hydrolysis, it was found that 35 mL of crude enzyme hydrolyzed 83.30% pectin from 45 mg of banana fiber at 15°C after 120 min of incubation, considered as the optimum level for hydrolysis of pectin from banana fiber at specified conditions. The diversion between the experimental values and predicted data of reducing sugar and the percentage of pectin yield were demonstrated through the points around the diagonal line (supplementary Figure 3).

**Figure 5:**
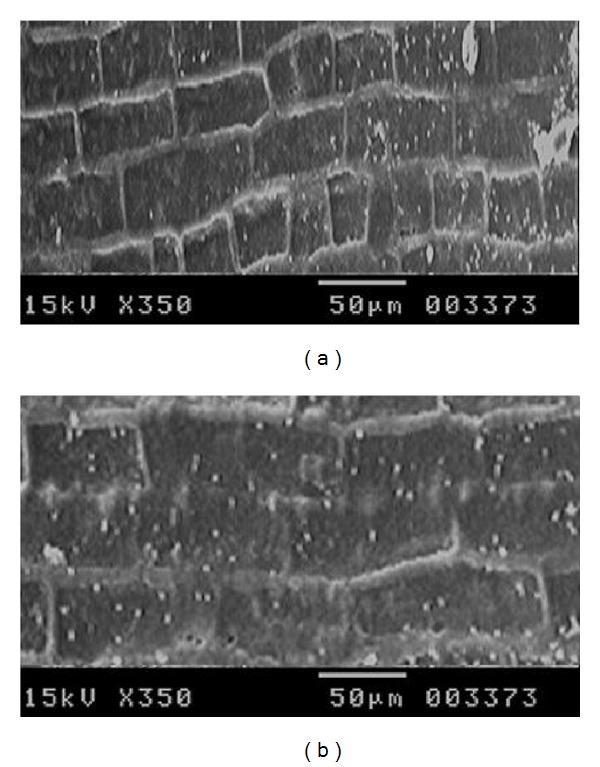
((a) and (b)) The scanning electron micrographs of banana fiber. Figures were taken before (a) and after (b) treatment with crude pectinase.

The treatment of banana fibers with crude enzyme obtained from *T. frigidphilosprofundus* was found to be successful. The scanning electron microscopic studies revealed the effectiveness of the process (Figure 5). The cell walls were demolished after the treatment with the crude enzyme and the cells were separated in the treated sample.

## 4. Discussion

Temperature is one of the major factors determining the distribution of microbial populations across the globe. According to Arnold et al. (2001), during the course of evolution, enzymes adjusted the strength and number of their stabilizing interactions to optimize the balance between rigidity (for stability) and flexibility (for activity) at their physiological and habitual temperatures [18]. The biocatalysts, produced by cold-adapted microorganisms which function under extreme cold condition and display a high specificity at low temperature, provide new opportunities for the potential application of these cold active enzymes in various industrial processes. To date, pectic enzymes from mesophilic and thermophilic microorganisms have been well studied, whereas the cold active pectinases from cold-adapted marine bacteria have not been studied extensively [1]. The clear zones, appeared around active colonies, indicated the utilization of pectin by* T. frigidphilosprofundus*. The efficiency of ruthenium red solution in detecting pectinolytic activity is well established [19]. Cloning of two pectatelyase genes from the marine Antarctic bacterium *Pseudoalteromonas haloplanktis* strain ANT/505 and characterization of the enzymes, reported by Truong et al, have shown the activity at low temperature [19] which is correlated with the present investigation. In the context of the study, we have classified cold active pectinase enzyme producing strain *T. frigidphilosprofundus* from a permanently cold environment such as the deep ocean. At the time of this paper preparation, it was asserted that no studies were found on pectinolytic activity from earlier reported *Thalassospira* species.

Recent works have focused on the halozymes from microorganisms [5]. This is the first report on the production of cold active halozyme PGase from deep marine bacteria. The strain was able to survive and produce PGase enzymes at high salt concentration. The temperature profile of the PGase indicates that this enzyme is efficient as well as useful at low temperatures. Regarding the pH stability of the enzyme, the maximum PGase activity of strain is at neutral pH; therefore this can be used for vegetable purees or other applications which need almost neutral pH range [20]. The stability of enzyme gradually reduced at higher pH [21, 22]. The acid tolerance property of the enzyme is a great advantage in pectin processing applications, since most fruit and vegetable tissues have acidic pH [23]. The maximum PGase activity was achieved using orange peel as the substrate under submerged culture condition. The work carried out by Cabeza et al. (2011) has found enzymatic activity at 30°C at pH 5.0. The concentrated extract presented good activity at 3°C, confirming that it was a cold-active enzyme [24]. Bajaj and Singhal demonstrated that addition of orange peel in the production media enhanced the PGase production in submerged and solid state fermentation [25]. PGase activity in cold adaptive *T. Frigidphilosprofundus* is found to be higher than that of the earlier reported mesophilic bacteria *Bacillus subtilis *[26], psychrophilic *Mucor flavus* [1], *Aspergillus niger* [19, 27], and thermophilic *Thermoascus aurantiacus* [25]. The influence of orange peel in the production media emphasizes the utilization of natural substrates, which is a waste material of the fruit juice industry. Therefore, our present results illustrate the possibility of using natural substrate for the production of PGase for industrial application.

Taking into account economic constraints and the possibility of large-scale production of PGase by psychrophilic *T. frigidphilosprofundus*, it is necessary to optimize the fermentation process using a medium with minimum and/or low-cost components without compromising the efficiency. The production was increased to 29 U/mL using RSM. With the combined application of ANN-GA, the PGase production was further enhanced significantly to 32 U/mL. As shown in this study, the PGase production was enhanced fourfolds than that of the earlier reported mesophilic and thermophilic bacteria, the point that makes *T. frigidphilosprofundus* an interesting strain for the production of cold active PGase [26, 28].

ANN coupled with GA has also been proven to be powerful tools in optimization and superior to statistical approaches such as RSM. Hence, it can be effectively used to represent the relationship between the input variables and PGase production. The *R* ^2^ (0.98) of ANN-GA justified an excellent correlation between the media components and PGase activity. The artificial neural network model fitted well with high statistical reliability and significance than CCD model (*R* ^2^ = 0.93). The approach presented in this paper can be employed for modeling and optimization of other bioprocesses.

In the paper industry, huge amount of chemicals are used for treatment of raw plant fibers which causes severe environmental threats and biological disturbances [28]. Therefore, enzymatic method is an alternate way to solve this problem. Pectinases are believed to be a permissive way in the processing of these fibers [29]. The banana fibers treated with crude pectinase obtained from *T. frigidphilosprofundus* were found to be successful. The catalytic activity at low temperature and maximum hydrolysis of the pectin in less incubation time indicate that PGase from *T. frigidphilosprofundus* would be a potential candidate for varied applications. The pectinase enzyme of *T. frigidphilosprofundus* showed more than 80% of pectin hydrolysis from banana fiber. In comparison with previous report, the present study has indicated potential uses of pectinase enzyme from *T. frigidphilosprofundus* in paper industry [30].

## 5. Conclusion

From the present investigation, it is clear that PGase from *T. frigidphilosprofundus* can be employed in higher pectinase enzyme production and potential application in pectin hydrolysis from banana fiber. In this study, the statistical approaches were applied for the enhanced production of cold active PGase. To date, no literature has been reported on the production and optimization of cold active PGase by a novel strain belongs to the genus *Thalassospira. *Therefore, this study can be employed for modeling and optimization of other bioprocesses. Thus, from these findings, it can be concluded that the psychrophilic bacteria *T. frigidphilosprofundus* isolated from the deep marine environment were better producers of PGase as compared to the reported mesophilic and thermophilic counter parts and in the near future this would be a potent source of cold active PGase enzyme.

## Supplementary Material

The influence and the interactions between various physico-chemical parameters in the production of polygalacturonase were represented in the supplementary figures. The supplementary figure 1 illustrates the common interaction plots of various medium components and their effect (response) on the production of polygalacturonase. The plots were generated from the Taguchi's L27OA design. The supplementary figure 2 illustrates the parity plot for the distribution of the prediction and experimental values of the PGase production. The supplementary figure 3 illustrate the parity plot for the distribution of the prediction and experimental values of the reducing sugar which is expressed in mg/ml and the pectin yield which is expressed in %.

## Figures and Tables

**Table 1:** Ranges and levels of the factors in coded form for L_27_OA.

Factors w/%	Symbol	Coded level		
		−1	0	1
Orange peel	*χ*_1_	0.25	0.50	1.0
Bactopeptone	*χ*_2_	0.2	0.3	0.5
MgCl_2_	*χ*_3_	0.3	0.59	0.75
Ferric Citrate	*χ*_4_	0.005	0.01	0.03
CaCl_2_	*χ*_5_	0.1	0.18	0.24
NaCl	*χ*_6_	0.8	1.2	1.4

**Table 2:** Design of Taguchi's L_27_OA and results for the activity of PGase.

Trial	*χ*_1_	*χ*_2_	*χ*_3_	*χ*_4_	*χ*_5_	*χ*_6_	^†^Response (U/mL)	Prediction (U/mL)
1	−1	−1	−1	−1	−1	−1	11.42 ± 0.18	11.40
2	−1	−1	−1	−1	0	0	11.32 ± 0.14	11.36
3	−1	−1	−1	−1	1	1	13.05 ± 0.09	13.00
4	−1	0	0	0	−1	−1	12.84 ± 0.19	12.89
5	−1	0	0	0	0	0	11.64 ± 0.07	11.59
6	−1	0	0	0	1	1	12.89 ± 0.15	12.92
7	−1	1	1	1	−1	−1	14.64 ± 0.23	14.62
8	−1	1	1	1	0	0	12.36 ± 0.07	12.38
9	−1	1	1	1	1	1	11.86 ± 0.18	11.90
10	0	−1	0	1	−1	0	13.00 ± 0.12	13.96
11	0	−1	0	1	0	1	15.52 ± 0.16	15.48
12	0	−1	0	1	1	−1	12.86 ± 0.09	12.84
13	0	0	1	−1	−1	0	11.15 ± 0.18	11.12
14	0	0	1	−1	0	1	14.86 ± 0.12	14.82
15	0	0	1	−1	1	−1	15.24 ± 0.18	15.14
16	0	1	−1	0	−1	0	15.08 ± 0.22	15.12
17	0	1	−1	0	0	1	13.74 ± 0.04	13.80
18	0	1	−1	0	1	−1	11.69 ± 0.16	11.71
19	1	−1	1	0	−1	1	12.42 ± 0.24	12.45
20	1	−1	1	0	0	0	12.79 ± 0.05	12.82
21	1	−1	1	0	1	−1	16.48 ± 0.12	16.52
22	1	0	−1	1	−1	1	14.00 ± 0.18	14.06
23	1	0	−1	1	0	0	15.65 ± 0.10	15.62
24	1	0	−1	1	1	−1	13.45 ± 0.12	13.48
25	1	1	0	−1	−1	1	13.37 ± 0.05	13.38
26	1	1	0	−1	0	0	11.56 ± 0.08	11.58
27	1	1	0	−1	1	−1	12.65 ± 0.15	12.64

^†^Experimental values are mean of triplicates test.

**Table 3:** Response table for signal to noise ratios and means.

Factors	*χ*_1_		*χ*_2_		*χ*_3_		*χ*_4_		*χ*_5_		*χ*_6_	
Levels	S/N	Mean	S/N	Mean	S/N	Mean	S/N	Mean	S/N	Mean	S/N	Mean
1	54.48	12.44	51.96	13.26	52.87	13.27	56.20	12.72	54.27	13.16	56.11	13.47
2	50.94	13.72	52.94	13.52	54.65	12.97	54.07	13.29	53.99	13.27	52.50	13.52
3	57.65	13.60	58.17	13.00	55.56	13.53	52.80	13.76	54.81	13.35	54.46	12.78
Delta	6.71	1.28	6.20	0.51	2.69	0.55	3.39	1.03	0.81	0.18	3.60	0.74
Rank	1	1	2	5	4	4	6	2	5	6	3	3

**Table 4:** Analysis of variance table for PGase activity.

Source	df	SS	MS	*F* ratio	*P* > *F*
Regression	6	57.019	57.019	155.25	0.005
Residual error	20	0.918	0.037		
Total	**26 **	**57.937 **			

*R*-Sq = 98.40% and *R*-Sq (adj) = 97.60%.

SS: sum of squares, MS: mean of squares, and df: degree of freedom.

**Table 5:** Ranges and levels of the factors for CCD matrix.

Factors w/%	Symbol	Coded level				
		−2	−1	0	1	2
Orange peel	*χ*_1_	0.50	0.75	1.00	1.25	1.50
Bactopeptone	*χ*_2_	0.10	0.15	0.20	0.25	0.30
MgCl_2_	*χ*_3_	0.65	0.70	0.75	0.80	0.90
NaCl	*χ*_6_	0.60	0.70	0.80	1.00	1.10

**Table 6:** Experimental design and results of PGase production using CCD.

Trial	*χ*_1_	*χ*_2_	*χ*_3_	*χ*_6_	^†^Response (U/mL)	CCD Predicated (U/mL)
1	−1.00	−1.00	−1.00	−1.00	25.08 ± 0.12	25.14
2	1.00	−1.00	−1.00	−1.00	23.11 ± 0.15	22.83
3	−1.00	1.00	−1.00	−1.00	25.29 ± 0.06	25.84
4	1.00	1.00	−1.00	−1.00	23.82 ± 0.18	23.96
5	−1.00	−1.00	1.00	−1.00	20.91 ± 0.21	20.93
6	1.00	−1.00	1.00	−1.00	23.25 ± 0.06	22.50
7	−1.00	1.00	1.00	−1.00	23.62 ± 0.12	23.70
8	1.00	1.00	1.00	−1.00	24.47 ± 0.15	25.70
9	−1.00	−1.00	−1.00	1.00	24.32 ± 0.18	23.66
10	1.00	−1.00	−1.00	1.00	21.36 ± 0.09	20.92
11	−1.00	1.00	−1.00	1.00	20.87 ± 0.08	20.29
12	1.00	1.00	−1.00	1.00	21.41 ± 0.16	21.96
13	−1.00	−1.00	1.00	1.00	19.78 ± 0.12	19.28
14	1.00	−1.00	1.00	1.00	28.08 ± 0.25	28.41
15	−1.00	1.00	1.00	1.00	21.09 ± 0.6	21.95
16	1.00	1.00	1.00	1.00	23.95 ± 0.16	23.52
17	−2.00	0.00	0.00	0.00	18.88 ± 0.09	18.59
18	2.00	0.00	0.00	0.00	17.76 ± 0.14	17.84
19	0.00	−2.00	0.00	0.00	23.11 ± 0.06	24.48
20	0.00	2.00	0.00	0.00	27.88 ± 0.12	23.27
21	0.00	0.00	−2.00	0.00	24.56 ± 0.14	24.51
22	0.00	0.00	2.00	0.00	21.02 ± 0.13	21.86
23	0.00	0.00	0.00	−2.00	25.61 ± 0.18	25.68
24	0.00	0.00	0.00	2.00	22.82 ± 0.06	22.03
25	0.00	0.00	0.00	0.00	24.52 ± 0.04	24.75
26	0.00	0.00	0.00	0.00	24.62 ± 0.09	24.75
27	0.00	0.00	0.00	0.00	24.52 ± 0.12	24.75
28	0.00	0.00	0.00	0.00	25.62 ± 0.06	24.75
29	0.00	0.00	0.00	0.00	24.62 ± 0.04	24.75
30	0.00	0.00	0.00	0.00	24.62 ± 0.12	24.75

^†^Experimental values are mean of triplicates test.

**Table 7:** Analysis of variance for quadratic model.

Source	df	Seq SS	Adj SS	MS	*F* value	Prob. (*P*) > *F *
Regression	14	159.379	159.3789	11.3842	16.45	0.000
Linear	4	53.139	53.1393	13.2848	19.20	0.000
Square	4	86.477	86.4770	21.6193	31.25	0.000
Interaction	6	19.763	19.7626	3.2938	4.76	0.007
Residual error	15	10.378	10.3782	0.6919		
Lack of fit	10	9.465	9.4649	0.9465	5.18	0.042
Pure error	5	0.913	0.9133	0.1827		
Total	29	169.757				

*S* = 0.8318, *R*-Sq = 93.90%, and *R*-Sq (adj) = 88.20%.

SS: sum of squares, MS: mean of squares, and df: degree of freedom.

**Table 8:** Model of coefficient estimated by multiple linear regression.

Source	Coefficient estimate	df	SE	Student's *t*-value	*P* value
Constant	24.753	1	0.3396	72.894	0.000
*χ*_1_	−0.185	1	0.1698	−1.092	0.292
*χ*_2_	0.952	1	0.1698	5.607	0.000
*χ*_3_	−0.662	1	0.1698	−3.899	0.001
*χ*_6_	−0.914	1	0.1698	−5.382	0.000
*χ*_1_^2^	−1.634	1	0.1588	−10.291	0.000
*χ*_2_^2^	0.408	1	0.1588	2.569	0.021
*χ*_3_^2^	−0.392	1	0.1588	−2.468	0.026
*χ*_6_^2^	−0.224	1	0.1588	−1.413	0.178
*χ*_1_ *χ*_2_	0.109	1	0.2079	0.526	0.607
*χ*_1_ *χ*_3_	0.971	1	0.2079	4.668	0.000
*χ*_1_ *χ*_6_	−0.107	1	0.2079	−0.514	0.615
*χ*_2_ *χ*_3_	0.517	1	0.2079	2.486	0.025
*χ*_2_ *χ*_6_	−0.023	1	0.2079	−0.111	0.913
*χ*_3_ *χ*_6_	−0.044	1	0.2079	−0.213	0.834

**Table 9:** Optimized medium composition using different methodologies.

Sl. no.	Concentration of component (w/%)						PGase production (U/mL)	
	*χ*_1_	*χ*_2_	*χ*_3_	*χ*_4_	*χ*_5_	*χ*_6_	Experiment	Predicated
1								
Before optimization	0.5	0.5	0.59	0.01	0.18	1.4	3.0	—
2								
L_27_OA	1.0	0.2	0.75	0.01	0.24	0.8	16.48 ± 0.12	16.52
3								
RSM	1.25	0.15	0.80	—	—	1.0	28.08 ± 0.25	28.41
4								
ANN-GA	0.75	0.20	0.86	—	—	1.1	31.47 ± 0.24	32.54

**Table:** (a)

Factors	Symbol	Coded level				
		−2	−1	0	1	2
Volume of crude enzyme extract (mL)	*A *	15	2025	35	40	
Temperature (°C)	*B *	10	15	20	25	30
Amount of banana fiber (mg)	*C *	25	3035	40	45	
Incubation time (min)	*D *	30	4590	120	150	

**Table:** (b)

Sl. No.	*A *	*B *	*C *	*D *	Pectin yield (%)		Reducing sugar (mg/mL)	
					^†^Experimental value^a^ CCD predicated		^†^Experimental value^b^ CCD predicated	
1	−1	−1	−1	−1	2.72 ± 0.16	2.67	0.03 ± 0.01	0.08
2	1	−1	−1	−1	1.63 ± 0.11	1.58	0.27 ± 0.04	0.24
3	−1	1	−1	−1	3.03 ± 0.32	2.98	0.09 ± 0.08	0.10
4	1	1	−1	−1	1.77 ± 0.13	1.73	0.19 ± 0.08	0.19
5	−1	−1	1	−1	3.05 ± 0.21	3.17	0.09 ± 0.04	0.05
6	1	−1	1	−1	2.02 ± 0.26	2.14	0.20 ± 0.07	0.18
7	−1	1	1	−1	3.94 ± 0.11	4.06	0.08 ± 0.03	0.06
8	1	1	1	−1	2.75 ± 0.16	2.87	0.12 ± 0.07	0.12
9	−1	−1	−1	1	3.12 ± 0.32	3.07	0.08 ± 0.04	0.08
10	1	−1	−1	1	1.74 ± 0.28	1.70	0.24 ± 0.09	0.24
11	−1	1	−1	1	3.73 ± 0.28	3.68	0.08 ± 0.06	0.08
12	1	1	−1	1	2.19 ± 0.16	2.14	0.14 ± 0.07	0.18
13	−1	−1	1	1	2.05 ± 0.27	2.17	0.17 ± 0.07	0.14
14	1	−1	1	1	0.84 ± 0.28	0.86	0.38 ± 0.03	0.38
15	−1	1	1	1	3.24 ± 0.21	3.36	0.11 ± 0.08	0.14
16	1	1	1	1	1.77 ± 0.13	1.89	0.29 ± 0.09	0.21
17	−2	0	0	0	4.46 ± 0.18	4.37	0.02 ± 0.00	0.01
18	2	0	0	0	1.90 ± 0.11	1.81	0.23 ± 0.05	0.24
19	0	−2	0	0	2.91 ± 0.18	2.82	0.09 ± 0.02	0.10
20	0	2	0	0	4.25 ± 0.21	4.16	0.05 ± 0.00	0.05
21	0	0	−2	0	1.38 ± 0.16	1.63	0.25 ± 0.07	0.19
22	0	0	2	0	2.29 ± 0.19	1.87	0.14 ± 0.02	0.19
23	0	0	0	−2	2.07 ± 0.16	1.98	0.16 ± 0.03	0.16
24	0	0	0	2	1.49 ± 0.13	1.40	0.25 ± 0.07	0.25
25	0	0	0	0	1.48 ± 0.18	1.48	0.10 ± 0.03	0.12
26	0	0	0	0	1.48 ± 0.14	1.48	0.15 ± 0.08	0.12
27	0	0	0	0	1.50 ± 0.19	1.48	0.10 ± 0.07	0.12
28	0	0	0	0	1.49 ± 0.24	1.48	0.10 ± 0.02	0.12
29	0	0	0	0	1.50 ± 0.34	1.48	0.14 ± 0.04	0.12
30	0	0	0	0	1.48 ± 0.15	1.48	0.10 ± 0.02	0.12

^a^ *R*-sq = 98.40% and ^b^ *R*-sq = 86.71%. ^†^Experimental values are mean of triplicates test.
