# Supplementary figures and images for: Optimization of Polygalacturonase Production from a Newly Isolated Thalassospira frigidphilosprofundus to Use in Pectin Hydrolysis: Statistical Approach
Source: Biomed Res Int. 2013 Dec 19;2013:750187. doi: 10.1155/2013/750187 (PMC3881631; doi:10.1155/2013/750187)

**Supplementary figures**


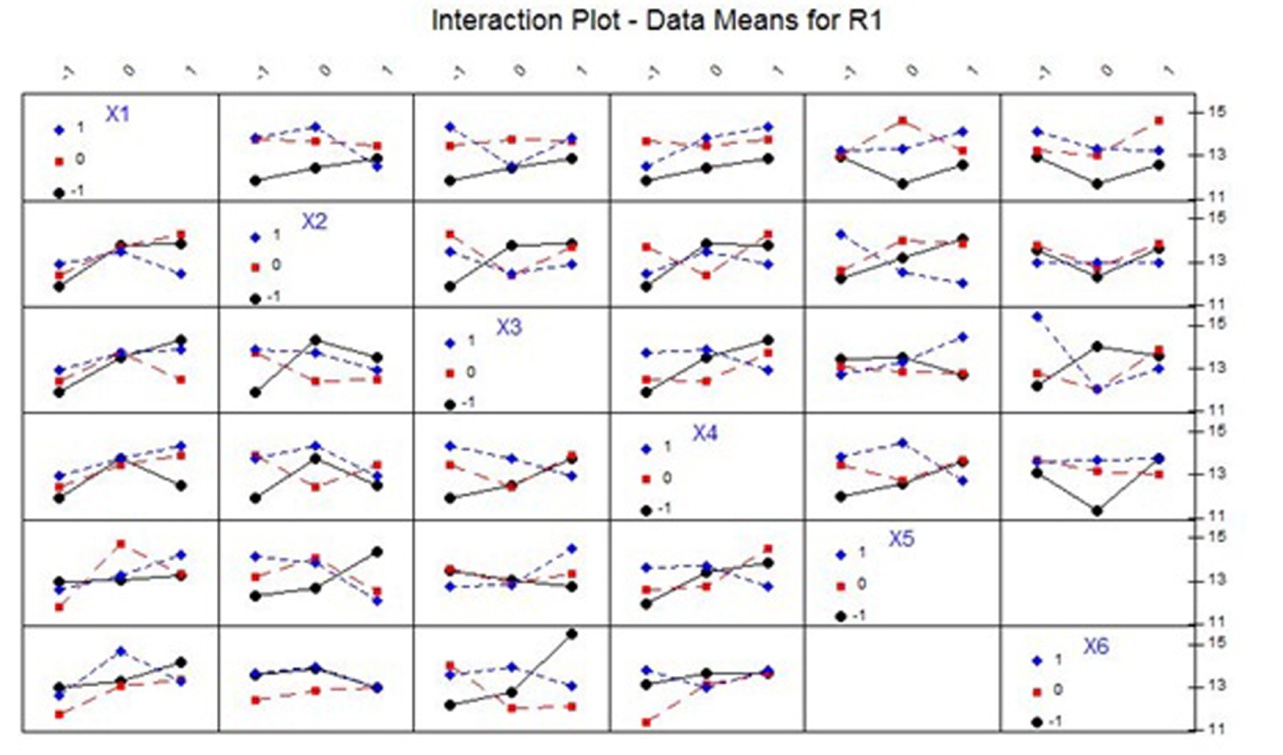


**Supplementary figure: 2**


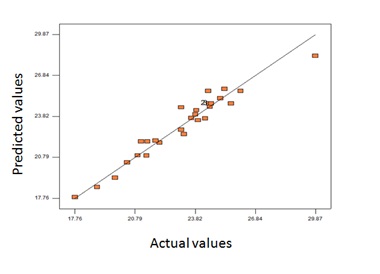


**Supplementary figure: 2**


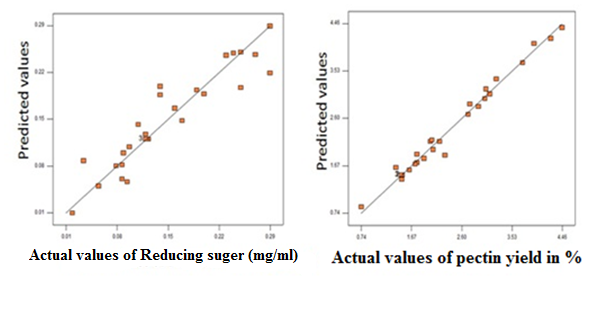


**Supplementary figure: 3**

Supplement: Supplementary file 1 — The influence and the interactions between various physico-chemical parameters in the production of polygalacturonase were represented in the supplementary figures. The supplementary figure 1 illustrates the common interaction plots of various medium components and their effect (response) on the production of polygalacturonase. The plots were generated from the Taguchi's L27OA design. The supplementary figure 2 illustrates the parity plot for the distribution of the prediction and experimental values of the PGase production. The supplementary figure 3 illustrate the parity plot for the distribution of the prediction and experimental values of the reducing sugar which is expressed in mg/ml and the pectin yield which is expressed in %. [file 750187.f1.docx]
